# Rice bean-adzuki bean multitrait near infrared reflectance spectroscopy prediction model: a rapid mining tool for trait-specific germplasm

**DOI:** 10.3389/fnut.2023.1224955

**Published:** 2023-12-15

**Authors:** Racheal John, Arti Bartwal, Christine Jeyaseelan, Paras Sharma, R Ananthan, Amit Kumar Singh, Mohar Singh, Gayacharan, Jai Chand Rana, Rakesh Bhardwaj

**Affiliations:** ^1^Amity Institute of Applied Science, Amity University, Noida, India; ^2^National Bureau of Plant Genetic Resources, Indian Council of Agricultural Research, Pusa, New Delhi, India; ^3^National Institute of Nutrition, Indian Council of Medical Research, Hyderabad, India; ^4^The Alliance of Bioversity International & CIAT – India Office, New Delhi, India; ^5^Germplasm Evaluation Division, National Bureau of Plant Genetic Resources, Indian Council of Agricultural Research (ICAR), New Delhi, India

**Keywords:** WMSC, weighted multiplicative scatter correction, SNV-DT standard normal variate-detrending, RPD, RSQ, coefficient of determination, minerals, regression

## Abstract

In the present era of climate change, underutilized crops such as rice beans and adzuki beans are gaining prominence to ensure food security due to their inherent potential to withstand extreme conditions and high nutritional value. These legumes are bestowed with higher nutritional attributes such as protein, fiber, vitamins, and minerals than other major legumes of the *Vigna* family. With the typical nutrient evaluation methods being expensive and time-consuming, non-invasive techniques such as near infrared reflectance spectroscopy (NIRS) combined with chemometrics have emerged as a better alternative. The present study aims to develop a combined NIRS prediction model for rice bean and adzuki bean flour samples to estimate total starch, protein, fat, sugars, phytate, dietary fiber, anthocyanin, minerals, and RGB value. We chose 20 morphometrically diverse accessions in each crop, of which fifteen were selected as the training set and five for validation of the NIRS prediction model. Each trait required a unique combination of derivatives, gaps, smoothening, and scatter correction techniques. The best-fit models were selected based on high RSQ and RPD values. High RSQ values of >0.9 were achieved for most of the studied parameters, indicating high-accuracy models except for minerals, fat, and phenol, which obtained RSQ <0.6 for the validation set. The generated models would facilitate the rapid nutritional exploitation of underutilized pulses such as adzuki and rice beans, showcasing their considerable potential to be functional foods for health promotion.

## 1 Introduction

Pulses are a profound source of protein, including essential amino acids, vitamins, minerals such as potassium and magnesium, and antioxidants. The carbohydrates in pulses include various key oligosaccharides, resistant starch, and dietary fiber, which are of great importance in promoting overall intestinal health (1). India is among the largest pulse producers in the world, covering approximately 29% of the world’s area under pulse production. It is also one of the largest pulse consumers, accounting for 19% of the world’s population. Chickpeas, pigeon peas, and mung beans are some of the major pulses grown in India (2). However, several pulse crops are yet to be explored and utilized to their full potential. These underutilized pulses, or “orphan crops,” are promising options due to their adaptability under adverse climatic conditions and resistance to pests and diseases (3). Underutilized pulses possess limited economic importance and lack formal seed distribution due to their growth under specific agroecological conditions but are of traditional importance to tribal communities. Cowpea, moth bean, horse gram, adzuki bean, and rice bean are some of the commonly underutilized pulse crops.

Rice bean and adzuki bean exhibit close similarity in pod and seed characteristics due to their similar evolutionary patterns (4). Adzuki bean is primarily cultivated in China, which is also considered its center of origin and harbors the largest collection of adzuki bean germplasm in the world. Apart from Chin, adzuki bean is largely grown in Japan and Korea as one of the important pulse crops (5). Along with its high protein, fiber, and carbohydrate content, adzuki bean is a good source of vitamins, such as thiamine, riboflavin, and niacin, and contains sufficient amounts of minerals, such as Fe, K, and Zn (6).

Rice bean is majorly grown in southern China, Nepal, northeast India, Bhutan, Indonesia, and Thailand. It is believed to have been domesticated from the wild cross-fertile type *Vigna umbellate* var. *gracilis* (7). Rice bean contains high protein content and is rich in tryptophan, methionine, and lysine. Additionally, the protein digestibility of rice beans is reportedly higher than that of many other pulses. Genomic studies indicate that apart from research on insect resistance and aluminum toxicity, not many studies have been done on rice beans to examine other nutritional qualities and traits (8).

To date, many advanced analytical techniques have been proposed and used for grain quality analysis, of which spectroscopy and computer vision are the most common non-invasive techniques. Spectroscopic techniques, including near infrared reflectance spectroscopy (NIRS), have been widely applied in the agricultural field to replace the time-consuming conventional analytical methods (9–11). The technique is based on the differential absorption of near-infrared wavelengths by molecules containing –C–H, –C–O–H, and –C–N–H bonds, which are the major NIR bands in biological materials. NIRS avoids the need for sample preparation and is non-destructive, rapid, economical, and time- and resource-saving.

The NIR spectrum is linked to the specific secondary characteristics of the samples, and the prediction models are built by developing regression equations between the spectral absorbance and laboratory analytical values (12). Before regression, the pre-processing of spectral data is of utmost importance to minimize the undesired variable effects, which are detrimental and interfere with quantitative analysis, leading to inaccurate results. These variations generally arise due to light scattering in the NIR region, resulting in non-linearity. An augmented pre-processing method can be used to correct the scattering effects of light by applying various pre-processing techniques, including derivatization, SNV (standard normal variate), normalization, detrending (DT), and MSC (multiplicative scatter correction). This can help enhance the validation of results and ensure accurate analysis. The multiplicative and additive effects are removed using spectral derivatives, in which the baseline effect is removed by the first derivative while the second derivative removes linear effects. The spectra are further refined by testing constant intervals of the spectral wavelengths (gap), followed by denoising of the spectra by first and second smoothing (13).

Previously, NIRS-based prediction models have been extensively developed for characterizing many crops such as maize, potato, cassava, rice, and pulses (14–19). NIRS models are a reliable technique for various biochemical estimations such as moisture, dietary fiber, ash, fatty acids, oils, protein, and sugar content with a minimum sample requirement (15, 20–23). Since these biochemical attributes determine the functionality of adzuki and rice bean germplasm, NIRS-based prediction modeling can be used for proximate analysis, and other constituents can contribute to the selection of the best crop varieties with a higher content of desired biochemical and nutritional constituents, such as protein, oil, fiber, minerals, and vitamins, accelerating the process of developing high-yielding varieties through breeding. Therefore, the present study aimed to develop combined prediction models for various biochemical parameters in adzuki and rice beans. Multiple chemometric combinations were used to build and select the best-fit model for each biochemical trait based on the comparison of lab analytical values and the NIRS spectra. The developed models would be useful for the screening and analysis of large samples of adzuki and rice beans.

## 2 Materials and method

### 2.1 Sample collection and preparation

**Figure 1:**
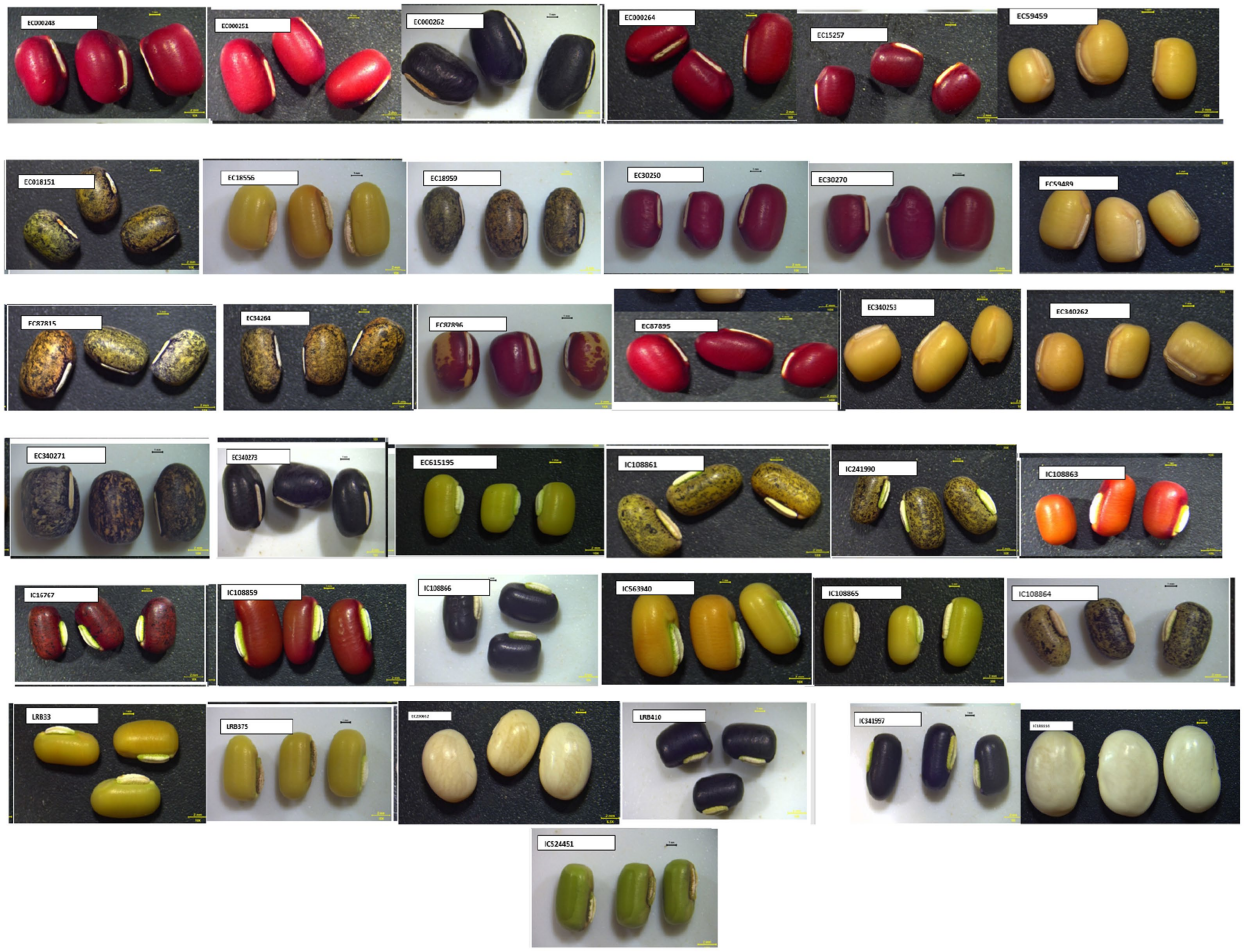
Morphologically diverse accessions of adzuki and rice beans.

Twenty indigenous and exotic accessions of rice bean and adzuki bean each (totaling 40) representing different shapes, sizes, and colors were collected from the ICAR-NBPGR Regional Station based in Shimla, Himachal Pradesh (India), accommodating morphological variability in both the pulses (Figure 1). The required quantity of the samples was ground, homogenized, and sieved through a 1 mm sieve on Foss Cyclotec^™^ 1093 Sample Mill (FOSS Analytical, Denmark) equipped with a grinding steel ring (Foss Mat: 10010233) to avoid any contamination while obtaining the flour of each sample. They were subsequently subjected to NIRS and wet lab analysis for biochemical parameters, namely total starch, protein, oil, dietary fiber, phenolics, sugars, antioxidant capacity, anthocyanin, and phytic acid.

### 2.2 Spectra acquisition

The homogenized samples were kept at room temperature (25°C) and were scanned on a FOSS NIRS 6500 spectrophotometer (Infrasoft International LLC, Port Matilda, PA, United States) to obtain the reflectance spectra. The reference cell (100% white mica) was scanned before each sample scan to ensure accuracy. Then, 5 g of the ground sample was loaded in the ring cup with a quartz window (internal diameter of 3.8 cm) and pressed slightly with a circular cardboard backing to ensure uniform packing. Each sample was scanned 32 times at 400–2,490 nm at 2 nm intervals, and an average spectrum was recorded for further analysis. The spectra were expressed as Log (1/*R*), where *R* is the respective reflectance. Post scanning, the moisture content of samples was estimated to be 9.2%–12.2% (average 11.2%) by AOAC 2005 method 934.01 (24).

### 2.3 Analysis of samples

Whole grains were evaluated for RGB value based on color comparison with the RHS color chart. All the adzuki and rice bean accessions were evaluated in the laboratory for total protein (AOAC 2001.11) (25). The total dietary fiber was estimated by using a Megazyme kit (K-TDFR-100A, Wicklow, Ireland) (AOAC method 985.29) (26). Total soluble sugars (27), starch (28), anthocyanin (29), and minerals were calculated using the Varian Fast Sequential AAS220 as per AOAC 985.35 method (30), while standard methods were followed for estimating phytate, phenols and antioxidant potential using Megazyme K-PHYT kit for phytates (31), Folin Ciocalteau reagent for total phenols (32), and CUPRAC and FRAP methods for antioxidant potential (33, 34). The total oil content was estimated in completely moisture-free, dehulled grain using pulsed NMR spectroscopy, which is based on the relaxation of protons when kept in an external magnetic field. Newport Analyzer Oxford 4000 and the standard operating protocol mentioned in the United States Department of Agriculture NMR Handbook were used (35).

### 2.4 Quality control

All the estimations were carried out in triplicate to ensure the reproducibility of the results. Suitable standards and reagent blanks were used to ensure accuracy during method validation and recovery checks for protein and TDF, using ASFRM-Rice-2 from PT-8 obtained from INMU, Thailand. For starch method validation, total starch control kit (K-TSCK) flours such as wheat starch and high amylose maize starch were used. The pulsed NMR-based total oil estimation method was validated using ISO10565:1998 and ISO10632:2000 standards for oilseed and their defatted residues. The instrument was calibrated three times for rice bean oil before the estimation to ensure the accuracy of the instrument. Oat flour control powder included in the Megazyme assay kit was used as a standard for the validation of the phytic acid estimation method.

### 2.5 Development of calibration equations

Out of the total 40 samples (20 each) of adzuki and rice beans, 30 (15 each) were used to develop the calibration (training) set, while the remaining 10 samples (5 each) were used in the validation (testing) set using the random selection method. Ensuring equal variability in both the calibration and validation sets justified the use of less number of samples for model development (36, 37). The calibration equations were developed on full-length spectra using the global equations program of Win ISI III project manager software version 1.50. Various combinations of pre-processing methods were used to optimize and extract the information from spectral data of adzuki and rice beans. The spectra were treated with many scatter correction methods, such as derivatization, SNV, WMSC, and SNV-DT.

The optimization of the calibration model was done by applying the 1st, 2nd, 3rd, and 4th derivatives combined with binning at different intervals of 4, 6, 8, 10, 12, 14, and 16 and smoothening by taking a moving average of 2, 4, and 6 points for each parameter under study. Following the spectral data pretreatment, laboratory and spectral data were regressed using the modified partial least-squares (mPLS) method, and the coefficient of regression (RSQ) was calculated. Each developed equation was tested on the validation set, and the best-fit calibration equation based on high RSQ showing a strong correlation between predicted and laboratory values was selected.

### 2.6 Statistical analyses

The statistical analyses were done to evaluate the coefficient of determination (RSQ), standard deviation (SD), standard error of calibration (SEC), standard error of prediction (SEP), ratio of performance deviation (RPD), bias and mean, using Win ISI^®^ III Project Manager software version 1.50 in cross-validation. The scatter plots were developed using MS Excel, while the histograms were developed using Jamovi statistical package version 2.4.1 (38). The comparison of means of various parameters was statistically tested using a paired sample *t*-test at a 95% confidence level using IBM^®^SPSS^®^ Modeler version 17 (39, 40).

## 3 Results and discussion

### 3.1 NIRS spectra

**Figure 2:**
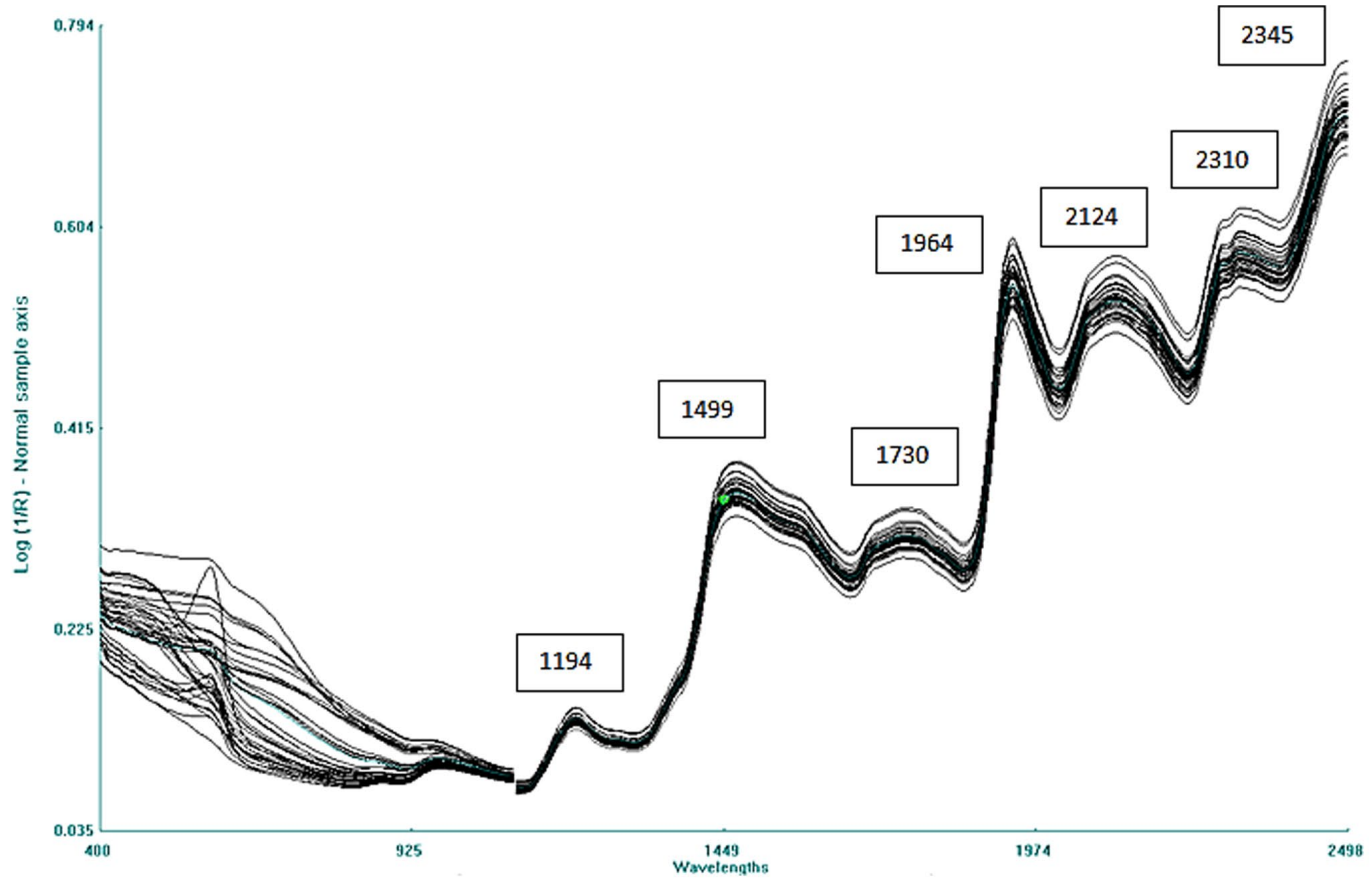
Combined NIRS spectra of 40 adzuki and rice bean germplasms.

The raw average spectrum of the combined 40 adzuki and rice bean accessions is given in Figure 2. The spectra consist of multiple overlapping bands with 7 major peaks at 1,194 nm related to C–H stretch second overtone, 1,499 nm due to O–H stretch second overtone, 1,730 nm due to C–H stretch first overtone, 1,964 nm due to O–H first overtone corresponding to moisture, and at 2,124, 2,310, and 2,345 nm due to C–H combinations or amide C–O stretch combination tones, respectively (41).

### 3.2 Biochemical estimation

**Figure 3:**
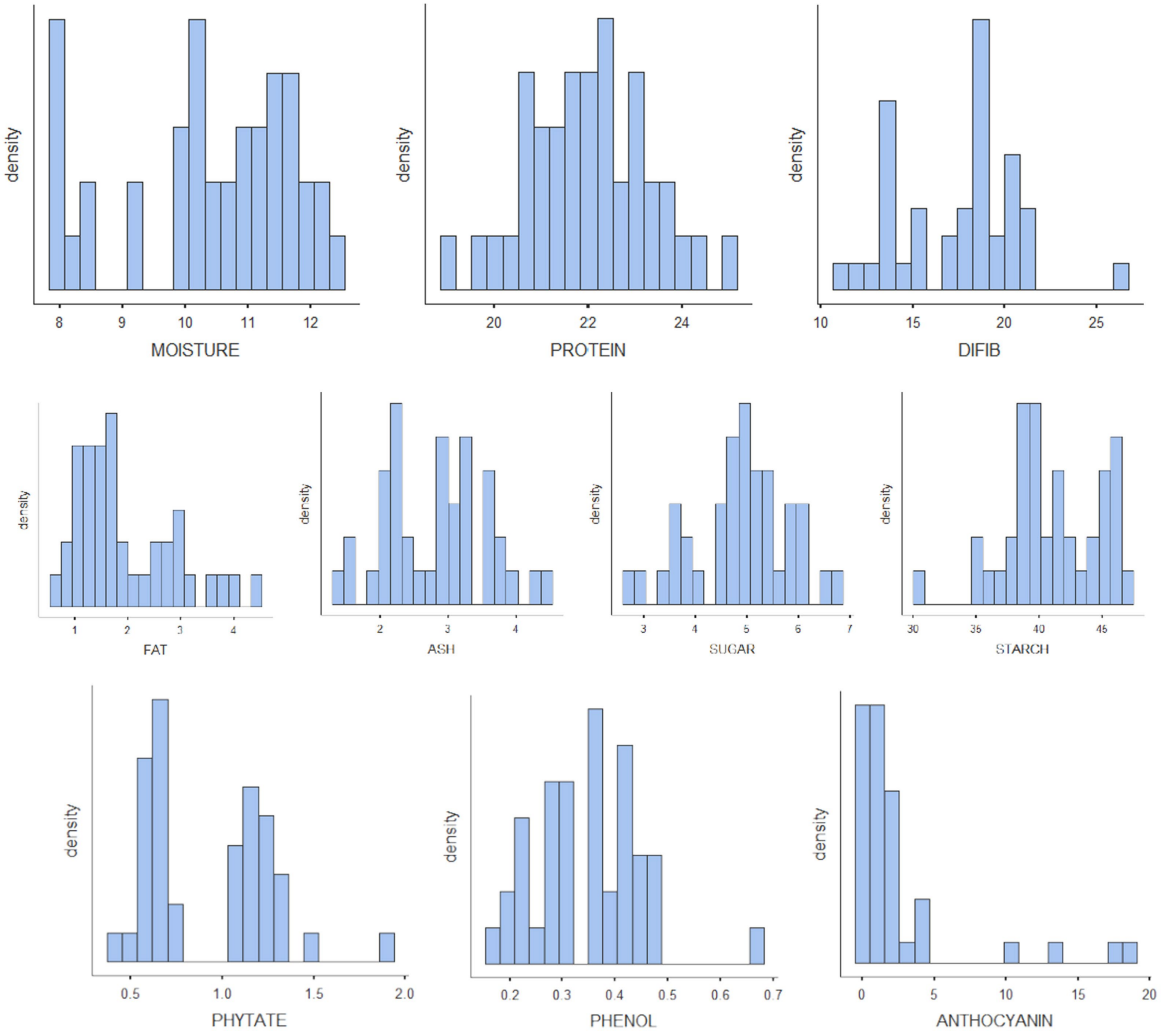

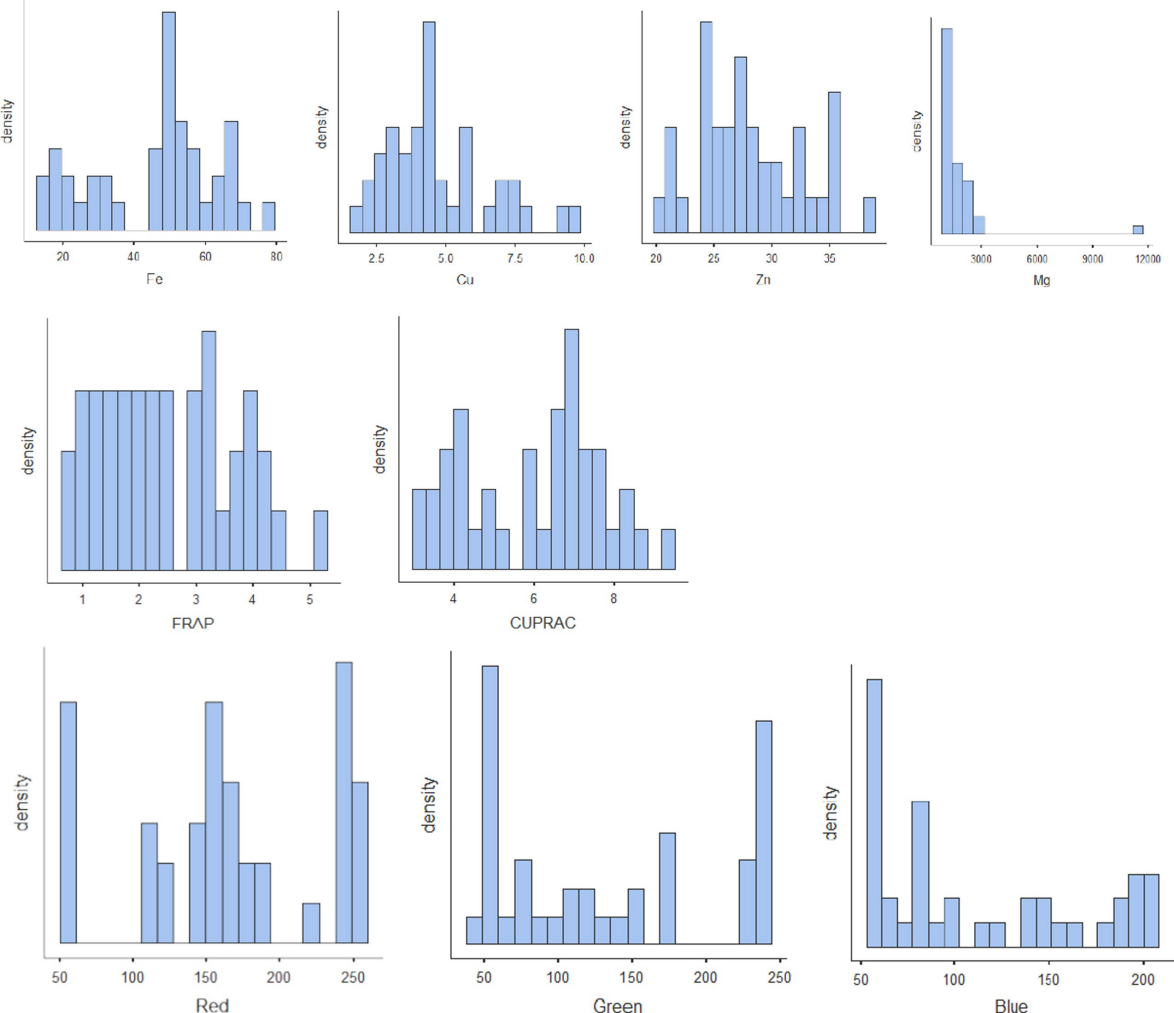
Histograms of all the nutritional parameters depicting the variability of the reference set.

The results of biochemical analyses for the generation of reference values are given in Tables 1A–D. All the values are expressed as mean ± SD. The moisture content ranged from 7.83% to 12.3% with mean ± standard deviation as 10.4 ± 1.37; protein, 19%–25% (22 ± 1.28); TDF, 11.1%–26.4% (17.4 ± 3.29); fat, 0.68%–4.47% (1.96 ± 0.958); ash, 1.32%–4.4% (2.8 ± 0.768); sugar, 2.76%–6.81% (4.89 ± 0.947); starch, 30.7%–47.3% (41.1 ± 3.75); phytate, 0.394%–1.88% (0.929 ± 0.346); phenol, 0.178%–0.68% (0.349 ± 0.101); anthocyanin, 0.117–18.7 (2.59 ± 4.43); FRAP, 0.62–5.06 GAE g/100 g (2.5 ± 1.16); and CUPRAC, 3.15–9.38 GAE g/100 g (6.01 ± 1.71). The mineral estimation for rice and adzuki beans ranged from 13.8 to 77.5 ppm (45.4 ± 17.6) for Fe, 1.92–9.79 ppm (4.69 ± 1.92) for Cu, and 20.3–38.5 ppm (28 ± 4.59) for Zn. The RGB value ranged from 54 to 253 (166 ± 64.7) for red, 41–236 (133 ± 73) for green, and 54–201 (111 ± 54) for blue. The results agreed with those reported by Shi et al. (42), Agarwal and Chauhan (43), and Sharma et al. (44). The variability of the data sets used for calibration is illustrated in the form of histograms in Figure 3. All the traits did not follow normal distribution, which is a desirable attribute in the case of prediction modeling for germplasm screening (16).

**Table 1:** Descriptive statistics of 40 rice bean and adzuki bean germplasm with **(A)** proximate composition, **(B)** antioxidants, anthocyanins, and phytates, **(C)** Mineral composition, and **(D)** RGB values.

A	Moisture	Protein	TDF	Fat	Ash	Sugar	Starch
*N*	40	40	40	40	40	40	40
Mean ± SD	10.4 ± 1.37	22 ± 1.28	17.4 ± 3.29	1.96 ± 0.958	2.81 ± 0.768	4.89 ± 0.947	41.1 ± 3.75
Range (%)	7.83–12.3	19–25	11.1–26.4	0.68–4.47	1.32–4.4	2.76–6.81	30.7–47.3

B	Phytate	Phenol	Anthocyanin	FRAP	CUPRAC
*N*	40	40	40	40	40
Mean ± SD	0.929 ± 0.346	0.349 ± 0.101	2.59 ± 4.43	2.5 ± 1.16	6.01 ± 1.92
Range (%)	0.394–1.88	0.178–0.68	0.117–18.7	0.62–5.06	3.15–9.38

C	Fe	Cu	Zn	Mg
*N*	40	40	40	40
Mean ± SD	45.4 ± 17.6	4.69 ± 1.92	28 ± 4.59	1,767 ± 1,612
Range (%)	13.8–77.5	1.92–9.79	20.3–38.5	884–11,162

D	Red	Green	Blue
*N*	40	40	40
Mean ± SD	166	133	111
Range (%)	54–253	41–236	54–201

### 3.3 Calibration and validation

**Figure 4:**
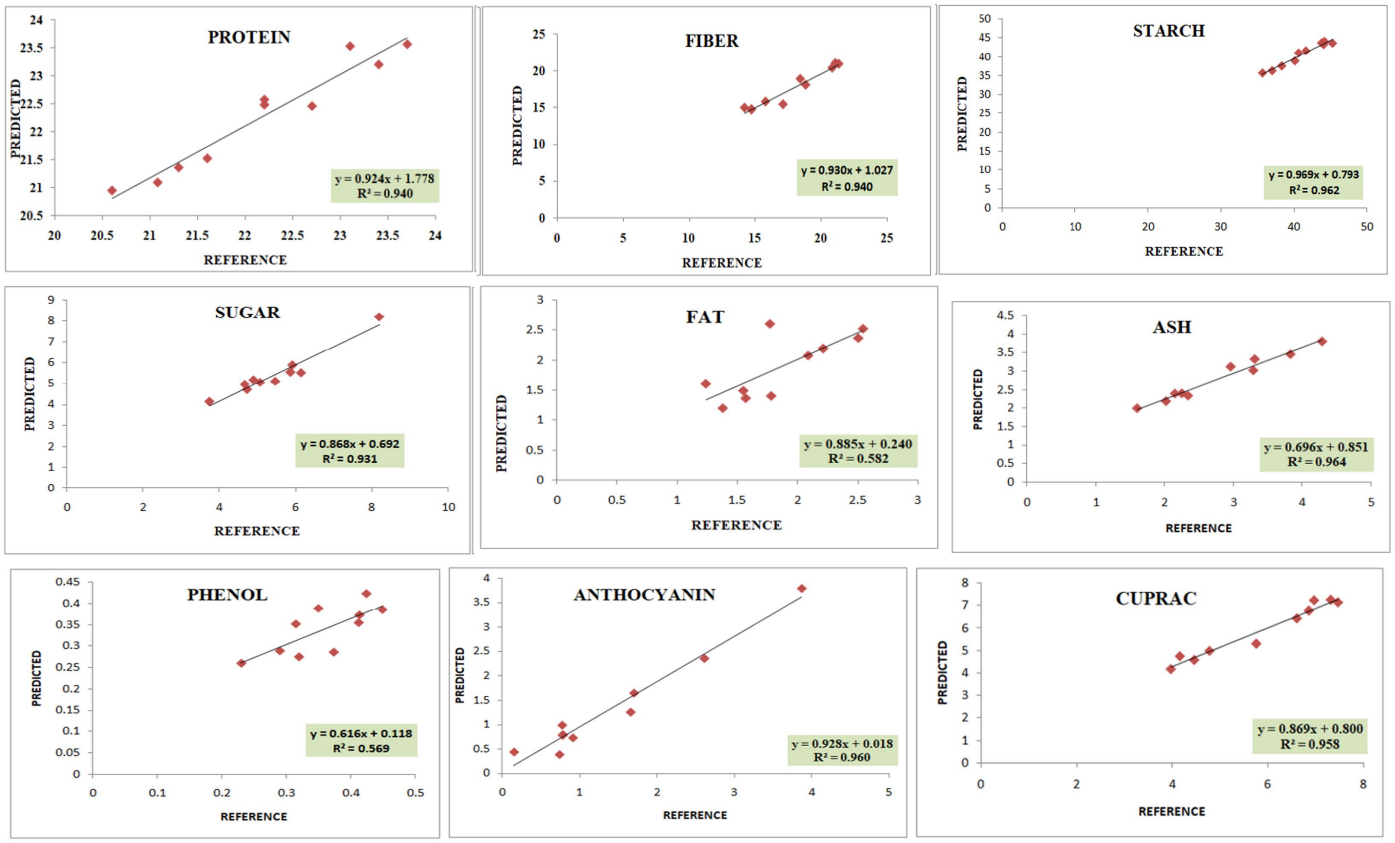

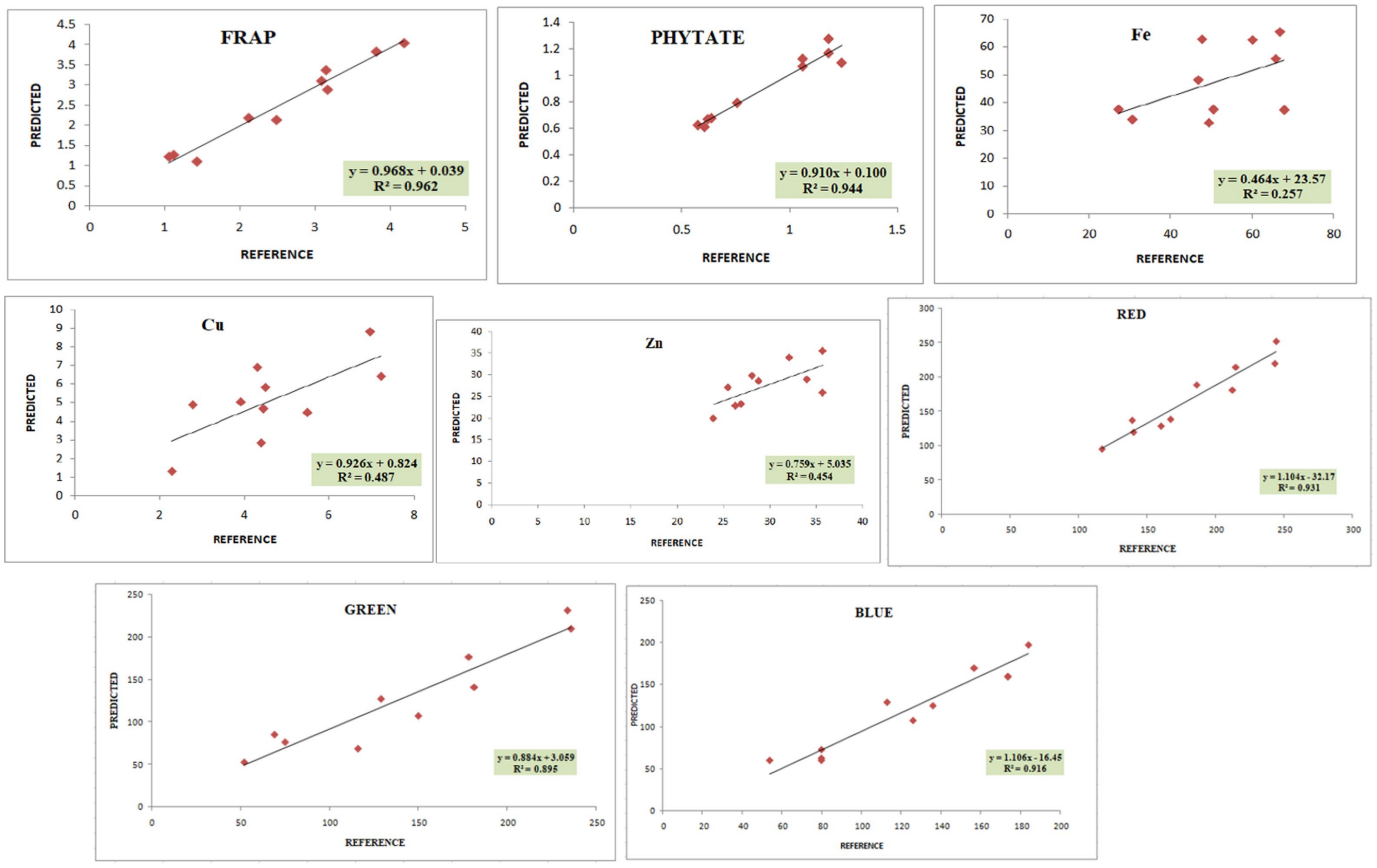
Validation plots of all the developed prediction models indicating linearity with coefficient of determination (*R*^2^).

The calibration and validation statistics are presented in Tables 2, 3, respectively. Scanning or analytical errors produce abrupt results for every trait; therefore, all the calibration equations were developed by removing 0–6 outliers during internal cross-validation (Table 2). Moreover, the removal of outliers in the validation step is a general practice to enhance external RSQ values. However, in our study, we achieved validation results without removing any outliers (Figure 4). The calibration models for different traits based on multiple chemometrics combinations resulted in calibration equations with varied levels of performance. The SD, SEC, SEP, and RPD values determined the usefulness of the NIR model. The calibration equation with an RPD value above 3 was considered highly useful, while the values lower than 2 depicted acceptable to poor model performance (45).

**Table 2:** Calibration model statistics for different parameters in the combined model for adzuki and rice bean genotypes by mPLS methods.

Parameters	Treatment	SEC	SEPC	SD	RSQ	No. of outliers
Protein	2,441	0.2154	0.197	1.218	0.974	1
Dietary fiber	3,661	1.48	1.358	3.196	0.821	2
Sugar	3,661	0.7249	0.555	0.674	0.337	6
Starch	3,661	1.56	1.521	3.686	0.83	2
Phytate	3,772	0.1172	0.113	0.331	0.884	1
Fat	3,661	0.795	0.685	1.064	0.602	2
CUPRAC	3,772	0.9102	0.869	1.888	0.788	1
Phenol	310,102	0.0707	0.059	0.114	0.731	1
FRAP	3,661	0.227	0.213	1.23	0.97	0
Anthocyanin	3,661	0.7623	0.803	5.024	0.974	0
Cu	3,772	0.871	0.821	1.806	0.793	2
Fe	3,661	11.89	9.72	17.14	0.678	3
Zn	2,441	3.94	3.532	4.379	0.35	4
Ash	2,441	0.3533	0.341	0.669	0.741	1
Red	3,661	14.02	24.98	70.23	0.874	0
Green	3,661	6.96	8.795	75.334	0.987	0
Blue	3,661	16.42	15.256	55.451	0.924	0

**Table 3:** Validation model statistics for parameters in the combined model for adzuki and rice bean genotypes by mPLS method.

Parameters	Math treatment	SEP	SD	RPD	RSQ ext.	*p*-value
Protein	2,441	0.312	1.374	4.4	0.941	0.681
Dietary fiber	3,661	0.993	2.571	2.6	0.941	0.828
Sugar	3,661	0.719	0.663	1.09	0.931	0.344
Starch	3,661	1.241	3.339	2.7	0.962	0.534
Phytate	3,772	0.058	0.28	4.8	0.945	0.827
Fat	3,661	0.557	0.617	1.1	0.583	0.027
CUPRAC	3,772	0.777	1.521	2.0	0.958	0.029
Phenol	310102	0.048	0.064	1.3	0.571	0.036
FRAP	3,661	0.972	1.72	1.8	0.963	0.087
Anthocyanin	3,661	0.791	1.39	1.8	0.960	0.893
Cu	3,772	0.48	1.256	2.6	0.469	0.243
Fe	3,661	9.816	15.99	1.6	0.257	0.366
Zn	2,441	1.875	3.092	1.6	0.454	0.335
Ash	2,441	0.44	0.534	1.2	0.965	0.065
Red	4,661	15.018	54.167	3.6	0.931	0.025
Green	4,661	23.044	66.48	2.61	0.895	0.103
Blue	4,661	14.601	48.185	2.92	0.917	0.119

#### 3.3.1 Dietary fiber and protein

In the present study, the best-fit model for dietary fiber was obtained using the 3rd derivative with a gap of 6 and simultaneous smoothening by taking a moving average of 6 data points. The mathematical treatment of 3,6,6,1 showed the highest RSQ value of 0.941 with RPD 2.6 using the mPLS method. The NIRS models for dietary fiber have seldom been reported in various crops, including pulses (46, 47). One of the earlier studies had reported the NIRS-based prediction model for dietary fiber with an acceptable RSQ value of 0.77 in chickpeas (48). The variability for dietary fiber content in our study ranged from 12.1%–26%, allowing a wide range of data for easy signal detection and model building. The development of a high-accuracy NIRS-based prediction model would prove to be rapid and cost-effective, considering the time and use of costly enzymes in the estimation process.

For protein, the best RSQ value of 0.941 was observed using the 2nd derivative with a gap and smoothing of 4 in treatment 2,4,4,1 with a very high RPD of 4.4. Previous works have also reported models using 1st and 2nd derivatives along with SNV-DT, SNV, and MSC (49, 50) and obtained RSQ values of 0.8 and RPD values of 2.3–2.4, indicating good model accuracy. The protein content in our samples ranged between 19% and 25%. This wide range in protein content, coupled with the highly polar behavior of amide bonds, allowed better learning even from a relatively small sample set, resulting in a high-accuracy model. Compared to both the above-mentioned studies, our results showed very high RSQ (0.952) and RPD (4.4) values, indicating higher reliability and applicability of the prediction model.

#### 3.3.2 Starch, sugar, and fat

Among different combinations tested, the regression equation with math treatment 3,6,6,1 using SNV-DT gave the best-fit model for starch with an RSQ value of 0.962 and RPD value of 2.7. The RSQ value of 0.962 with low error is achieved due to the wide range of variability of 30%–44.9% in training data, and precision in prediction is indicated by the RPD value. Therefore, the results based on the statistical values suggest good model performance for screening and quantitative analysis of total starch content in pulses. Limited mathematical models for starch are reported in pulses to date, including common bean (RSQ 0.5, RSQ 0.88), faba bean (RSQ 0.93), and pea (RSQ 0.80) (50–53).

The high-accuracy prediction model for sugar was observed using the mathematical treatment of 3,6,6,1 using scatter correction by the SNV-DT method. A high correlation between the predicted and laboratory values of the validation set was obtained, indicated by an RSQ value of 0.931; however, the model qualifies only for use in screening as a low RPD value of 1.09 was obtained. Previously, a prediction model for sugar had been developed and reported based on an NIRS study of 733 chickpea germplasms with a correlation coefficient of 0.87, which is comparable to the result of the present study (54).

The fat content in rice and adzuki bean accessions ranged from 0.68% to 4.47%. The developed prediction model for fat in the present study with an RSQ value of 0.583 and RPD value of 1.1 was obtained with treatment 3,6,6,1. The model statistics indicate that a positive correlation exists between spectra and actual fat content. In addition, a RPD value of >1 indicates the model usage for preliminary screening purposes (55). Many models have been reported for predicting fat with varying degrees of RSQ values in different leguminous crops, such as soybean (0.4), common bean (0.77), and chickpea (0.9) (54, 56, 57). The lower RSQ in our study is due to the limited sample size and low range of variability in fat content. The overall lower concentration of fat in each accession is another possible reason for the low RSQ values.

#### 3.3.3 Phenol, CUPRAC, FRAP, and anthocyanin

The best model for phenol was identified after model validation with treatment 3, 10,10,2 coupled with WMSC scatter correction, giving an RSQ value of 0.571 and RPD of 1.3. Very low RSQ values were obtained by using the gap <10 and with 1st, 2nd, and 4th order derivatives using the SNV-DT scatter correction method (data not shown). The combinations of derivatives and gaps by applying WMSC also failed to give the desired results of higher RSQ for high model accuracy. The narrow range of 0.19%–0.47% of phenols recorded for the reference dataset was not sufficient for improved statistical values. Therefore, the model lacked usability with respect to accuracy and reliability. However, previously, the SNV method has proved to be useful in building the prediction model for phenol using 60 different genotypes of mung bean with RSQ = 0.987, suggesting high accuracy (58). The mPLS and PLS methods have been previously used for developing prediction models for phenolics and anthocyanin in various crop plants with acceptable RSQ values (59, 60).

The models for the estimation of antioxidative capacity by CUPRAC and FRAP were built using treatments 3,7,7,2 and 3,6,6,1 with WMSC. The RSQ value for antioxidative capacity by CUPRAC (values ranging from 3.1% to 9.38%) under treatment 3,7,7,2 was 0.958 with an RPD of 2, while by FRAP (values ranging from 0.62% to 5.06%), best RSQ value was observed under treatment 3,6,6,1 with 0.963 and RPD value of 1.8.

The variability range of 0.08%–1.6% was observed among 40 different genotypes for anthocyanin content. The highest RPD value of 1.8 was observed for anthocyanin, with a good RSQ value of 0.960 under treatment 3,6,6,1. However, the reason for the low RPD value can be attributed to the lower anthocyanin concentration in adzuki and rice beans. Based on the RSQ value and other statistical parameters (Supplementary Tables S2, S3; validation), the model shows good prediction accuracy for screening pulse germplasm. Spectroscopy-based prediction models for various phytochemicals, including phenols and individual antioxidants, have been found (60, 61), but no literature was traced for estimating the total anthocyanin content in adzuki and rice beans through NIRS modeling. This may be the first report on a NIRS-based prediction model for anthocyanin in pulses with high accuracy and reliability.

#### 3.3.4 Phytate, minerals, and ash

Treatment 3,7,7,2 gave the best RSQ of 0.945 for phytate with an RPD value of 4.8 by the mPLS method. In the case of phytate calibration, the gaps were increased to 7 with subsequent smoothing of 7 and 2 in combination with the 3rd derivative using WMSC. The variability range for phytate in different accessions of adzuki and rice beans varied between 0.39%–1.88%, which allowed less error and increased reliability of the regression model based on RSQ and RPD within the given range of data set. An RPD value of 4.8 has not yet been reported for phytate models in pulses. The high coefficients of determinations for phytate validation were possible to achieve because of the highly polar behavior of the phytate molecule. Apart from its limited beneficial role as stored phosphorus, phytate is one of the major antinutrient factors present in pulses, thus limiting their nutritional availability. Estimation of phytate in pulses would allow the selection of the pulse varieties with lower phytate content, making them preferable over others. Our model promises high accuracy for prediction purposes, as evidenced by the high RSQ along with significantly high RPD. A previous study developed a prediction model for phytate in common bean with an RSQ value of 0.88 using the 1st derivative by the PLS method (60). However, in our study, we have tried higher-order derivatives for enhanced feature extraction, gaps, and smoothing, which altogether performed excellent spectral refinement, making the currently developed model more applicable than the previously reported models.

The 3rd derivative, along with gap and smoothening of 6, 1, gave the best correlation values for Fe with RSQ = 0.257 and RPD = 1.6. Among all the possible combinations, treatment 3,7,7,2 gave the best RSQ = 0.487 with RPD = 2.6 for Cu, and for Zn, 2,4,4,1 gave RSQ =0.454 with RPD = 1.6 indicating the best combinations of derivative, gap, and smoothening. For scatter correction, SNV-DT was employed for Fe, while for Cu and Zn, WMSC gave the best-fit results. Although the combined model for rice and adzuki beans for these elements has not been reported so far, the present study shows low model accuracy for Fe as well as for Cu with comparatively poor RSQ values using the mPLS method. An important reason behind the failure of developing calibration for microelements could be due to the poor absorption of energy by minerals in the NIR region because they are present in bound form with organic molecules rather than in a free form, which can result in poor absorption (57).

The best model for ash content was built using the 2nd derivative with a gap and smoothing of 4 with the treatment 2,4,4,1. The RSQ value for validation was 0.654, and the RPD value was 1.2 using the mPLS method with WMSC for scatter correction. The ash content among all the tested accessions was between 1.32% and 4.40%. Previous studies on soybean and chickpea genotypes reported RSQ values of 0.6 and 0.7 for the prediction model of ash content, which is comparable to the results of the present study (Ferreira et al., 2013; Flinn et al., 1998). The RSQ and RPD in the case of ash content are obtained for individual minerals. The NIRS works on stretching and bending of bonds, which is not possible for mineral ions as they exist in combination with organic molecules with an indirect relationship. The lower RSQ of ash is indicative of the existence of the non-polar/non-organic nature of ash on sample combustion, due to which the bending and stretching of bonds do not apply values (62).

#### 3.3.5 RGB values based on the RHS chart

The identification and quantification of color is important as it is not only related to consumer preferences but is an indication of the absence or presence of certain phytochemicals such as anthocyanins, carotenoids, and phenols. A quantification of these attributes is useful to maintain uniformity of seed lots for color, quality control of processed foods, and ascertaining the price. The mPLS-based prediction model using treatment 4,6,6,1 was developed on whole grain spectra for RGB values based on the RHS color chart and RSQ values of 0.931, 0.895, and 0.917 for red, green, and blue colors, respectively, were obtained with an RPD range of 2.92–3.6.

While only limited pre-processing methods have been used and reported in previous studies (15), we tested various combinations of chemometric parameters with four different derivatives to develop the best-fit model for all parameters. The spectral resolution was enhanced by removing noise on the application, mostly by the 2nd and 3rd-order derivatives for tested parameters. The effect of related co-variates on each other was minimized or eliminated by adjusting the data points by using the required gap, while the background noise was further lowered using smoothing. Merely increasing the derivative was not sufficient to obtain the required model accuracy; hence, the data points were lowered to find more refined information and develop the model.

The reduced data points represented as a gap indicate the exclusion of the effect of multi-collinearity, which is the effect of one latent variable on the other in the regression model. Multi-collinearity among the variables reduces the accuracy of the estimated coefficient, thus lowering the accuracy of the regression model. Therefore, increasing the gap and thus reducing the interdependent effect of variables resulted in better RSQ for certain parameters. The effect of multi-collinearity sometimes also arises due to the duplication of the same variables. Therefore, to avoid confusion, combinations of derivatives, spectral refinement, and gaps for removing the undesired noise and possible erroneous data recordings were used. In the case of some parameters such as fat, phenol, and ash, the lower RSQ value correlated to the use of a smaller sample size and the limited range of variability in data. Therefore, for a more reliable model with high accuracy for the above-mentioned parameters, there is a requirement for a large sample size. The model prediction accuracy and applicability are represented by RSQ and RPD. Higher numbers of latent variables result in an over-fit of the model due to their effect on each other, resulting in high bias, while too low numbers of variables result in an under-fit model with lower bias (63, 64).

In our study, the WMSC method gave better RSQ and RPD values for certain parameters, while best-fit models for other parameters were obtained using SNV-DT. In parameters with SNV-DT, the particle size effect, and scattering were removed using SNV, while the variation in baseline shift was corrected by detrending. Both SNV-DT and MSC (multiplicative scatter correction) have been widely used to reduce spectral variance due to the particle size effect. Fontaine et al. (65) developed and reported a prediction model for amino acids in cereal and sorghum genotypes where spectra were treated with SNV-DT and gave a more accurate model by reducing spectral noise for amino acids in test samples. However, the application of MSC enhanced the accuracy of the model for amino acids in soybean samples with an RSQ value of 0.91 (66).

The scatter correction by WMSC gave better RSQ values compared to the SNV-DT due to reduced baseline and multiplicative effects. Also, WMSC produces outliers in the score plots obtained by the spectral treatment, while SNV only induces curved structures of these score plots. Elimination of outliers thus gives better RSQ values (67). The best model with high accuracy employing all the parameters mentioned is thus selected based on the values of RSQ and RPD. Therefore, in the present study, the best model was selected by trial and error with applicable values of RSQ and RPD for each parameter under study. Based on the RSQ and RPD values, the models for different parameters could be used for screening of large samples and rapid quantification of adzuki and rice bean germplasms as well as other related pulses. The statistical analyses using SPSS showed no significant difference between predicted and laboratory values, with *p*-values higher than 0.05 indicating model acceptability.

## 4 Conclusion

The present study developed various mathematical equations that can be employed in screening and for the prediction of quantitative values for different biochemical parameters in adzuki and rice bean species. These combined models can also be used to anticipate the values of different biochemical constituents in similar pulses. The models can be employed for developing scanning devices to know the actual content of these parameters for consumer satisfaction in supermarkets. Thereafter, the pulses can be assorted and labeled accordingly for varieties rich in specific nutrient or antinutrient factors.

While these combined models are truly functional for the prediction of many biochemical components, we could not achieve the desired RSQ values for sugars and phenols. There is scope for refinement by including a more diverse sample set (location, season, etc.) for improved calibrations of such traits. Parameters such as phenols, phytates, and antioxidants could be estimated with HPLC to generate robust reference values. Investigating and optimizing advanced chemometrics, non-linear regressions, and machine learning algorithms such as artificial neural networks (ANN) and inverted partial least squares (iPLS) could improve the modeling process. Additionally, the prediction models could be developed for whole grain adzuki and rice bean germplasms, which would completely make the process non-destructible.

## Data availability statement

The raw data supporting the conclusions of this article will be made available by the authors, without undue reservation.

## Author contributions

RJ: investigation and NIR model refinement. AB: writing original draft manuscript. CJ, PS, and RA: inter-laboratory method validation of wet lab data and manuscript review. AS, MS, and Gayacharan: review and revisions. JR: provided diverse germplasm accessions, conceptualization, and critical review. RB: study plan, coordination, NIR modelling, and review of results and editing. All authors contributed to the article and approved the submitted version.
