# Supplementary figures and images for: Rice bean-adzuki bean multitrait near infrared reflectance spectroscopy prediction model: a rapid mining tool for trait-specific germplasm
Source: Front Nutr. 2023 Dec 15;10:1224955. doi: 10.3389/fnut.2023.1224955 (PMC10757333; doi:10.3389/fnut.2023.1224955)

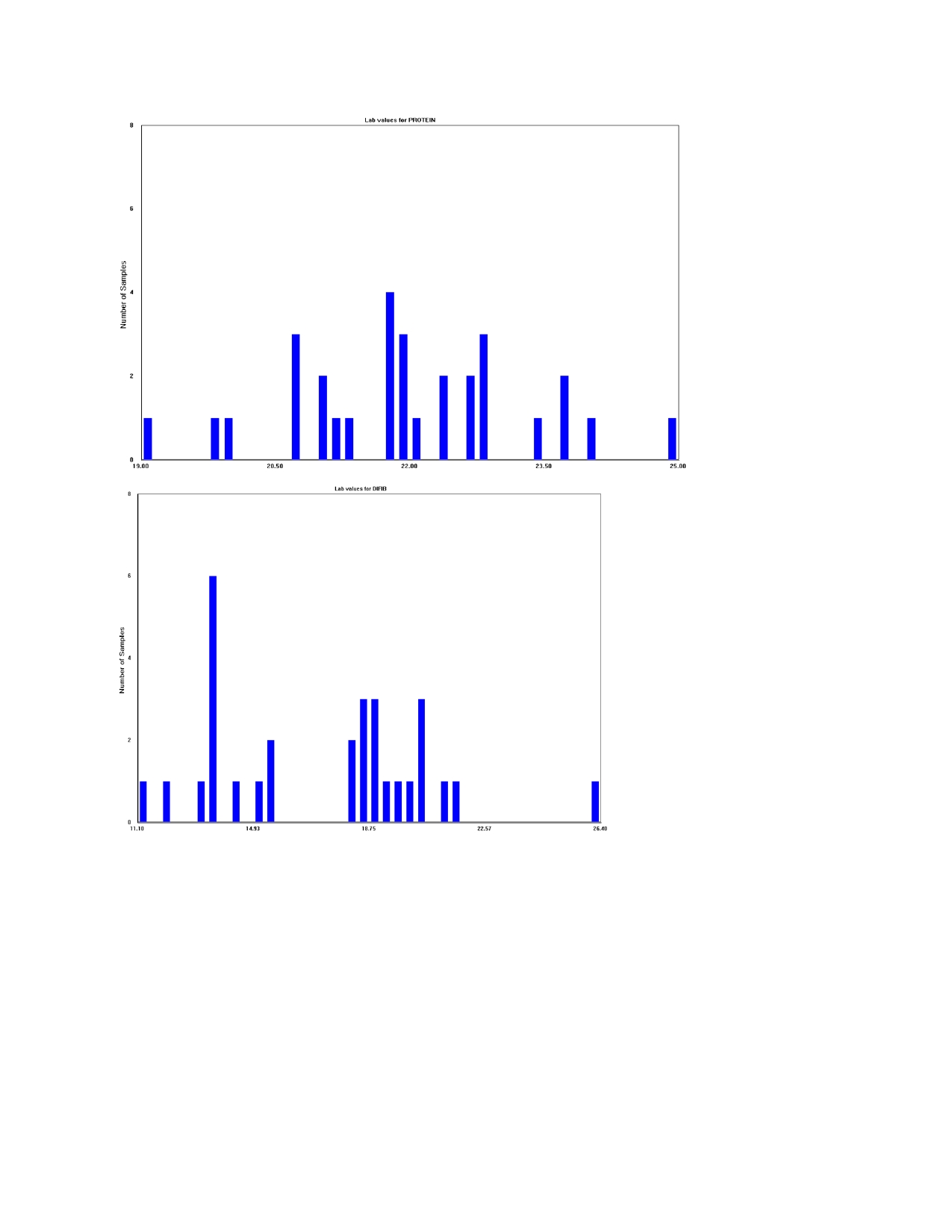

Supplement: Supplementary file 1 [file Data_Sheet_1.ZIP › page0001.jpg]

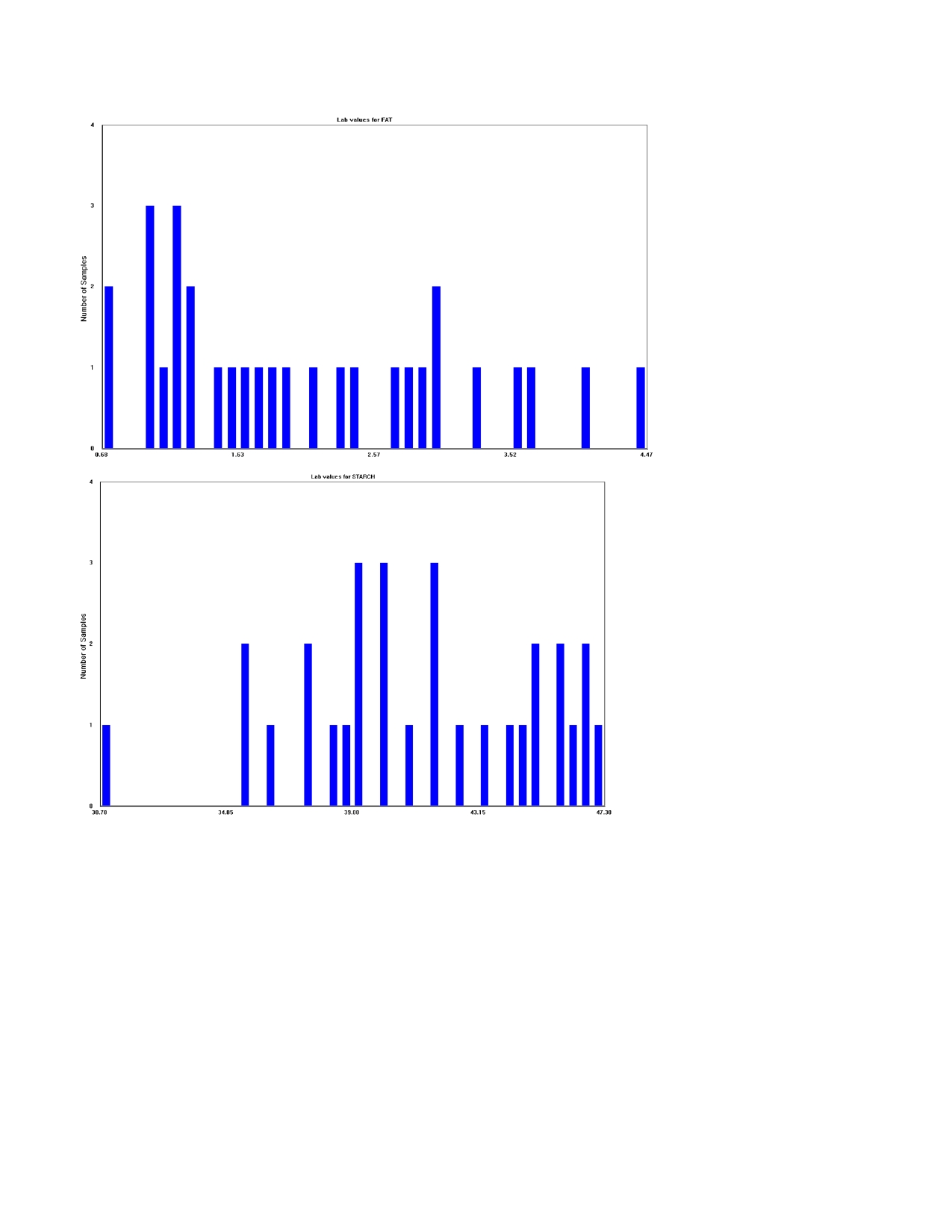

Supplement: Supplementary file 1 [file Data_Sheet_1.ZIP › page0002.jpg]

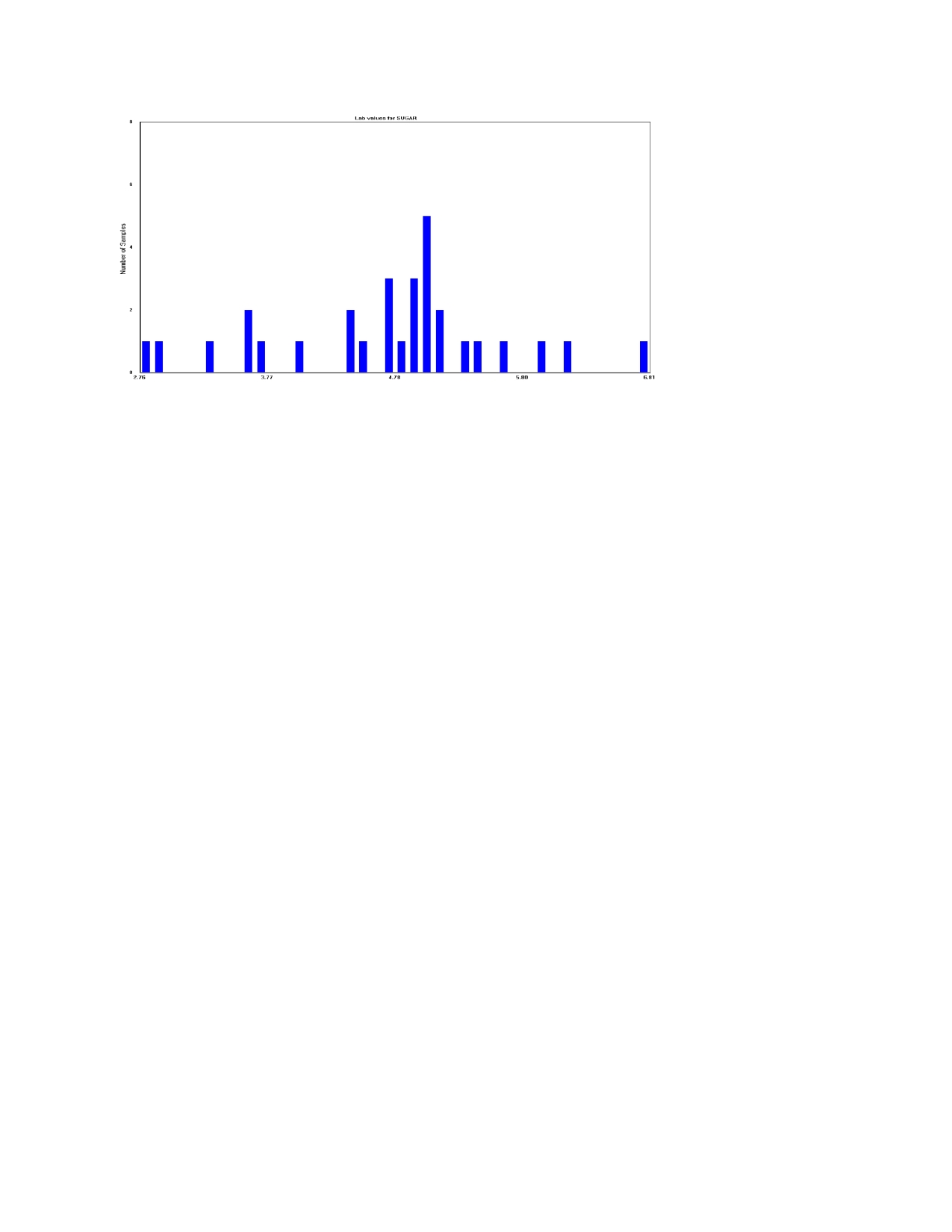

Supplement: Supplementary file 1 [file Data_Sheet_1.ZIP › page0003.jpg]

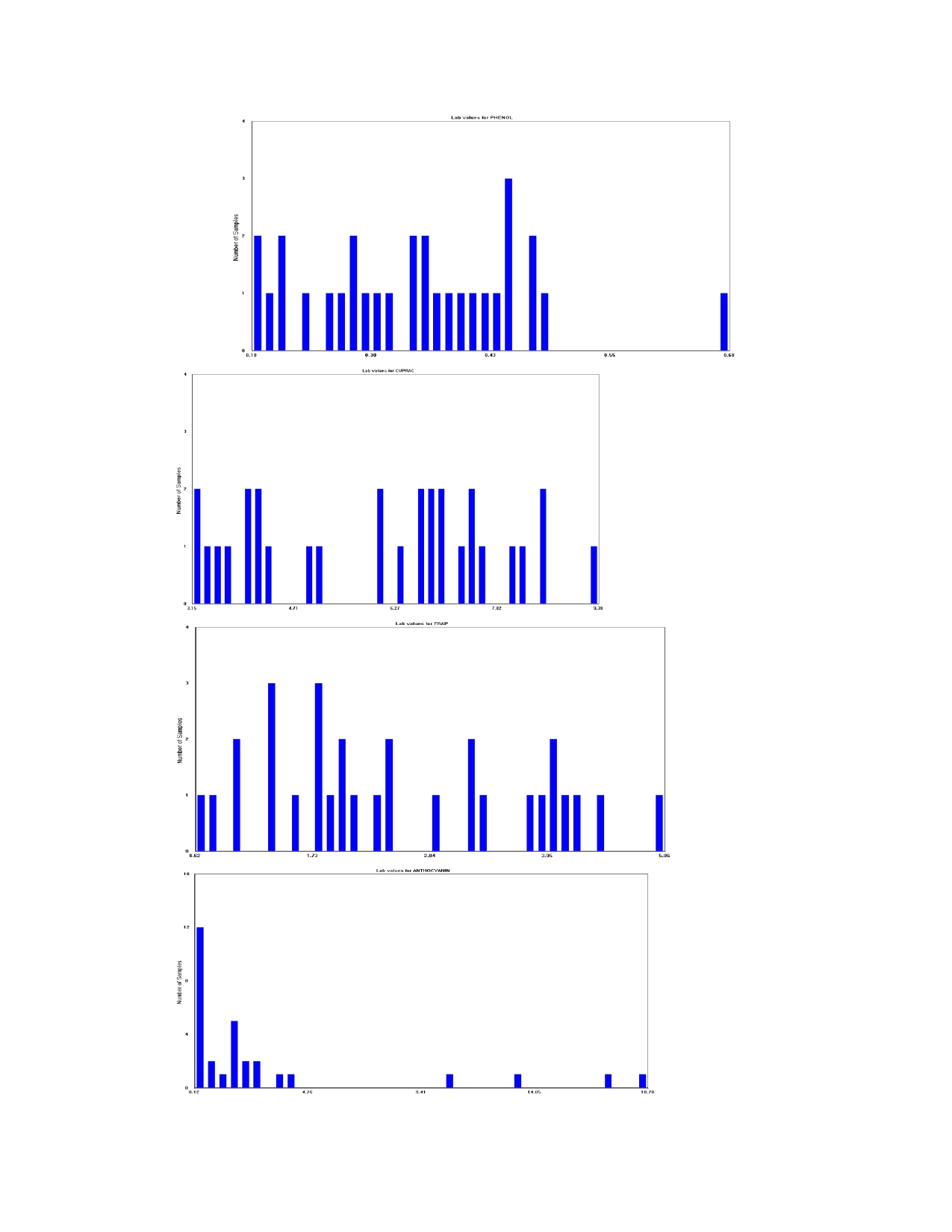

Supplement: Supplementary file 1 [file Data_Sheet_1.ZIP › page0004.jpg]

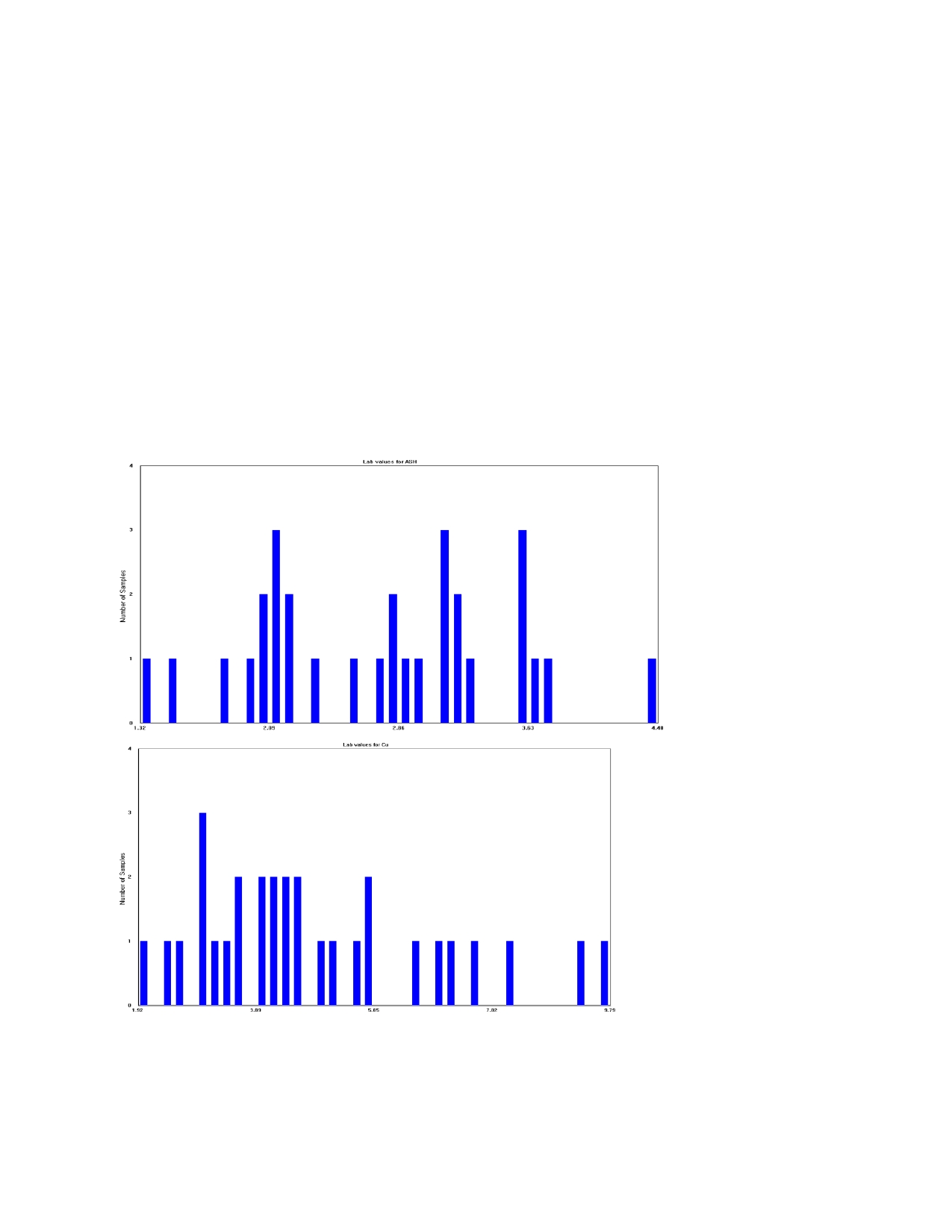

Supplement: Supplementary file 1 [file Data_Sheet_1.ZIP › page0005.jpg]

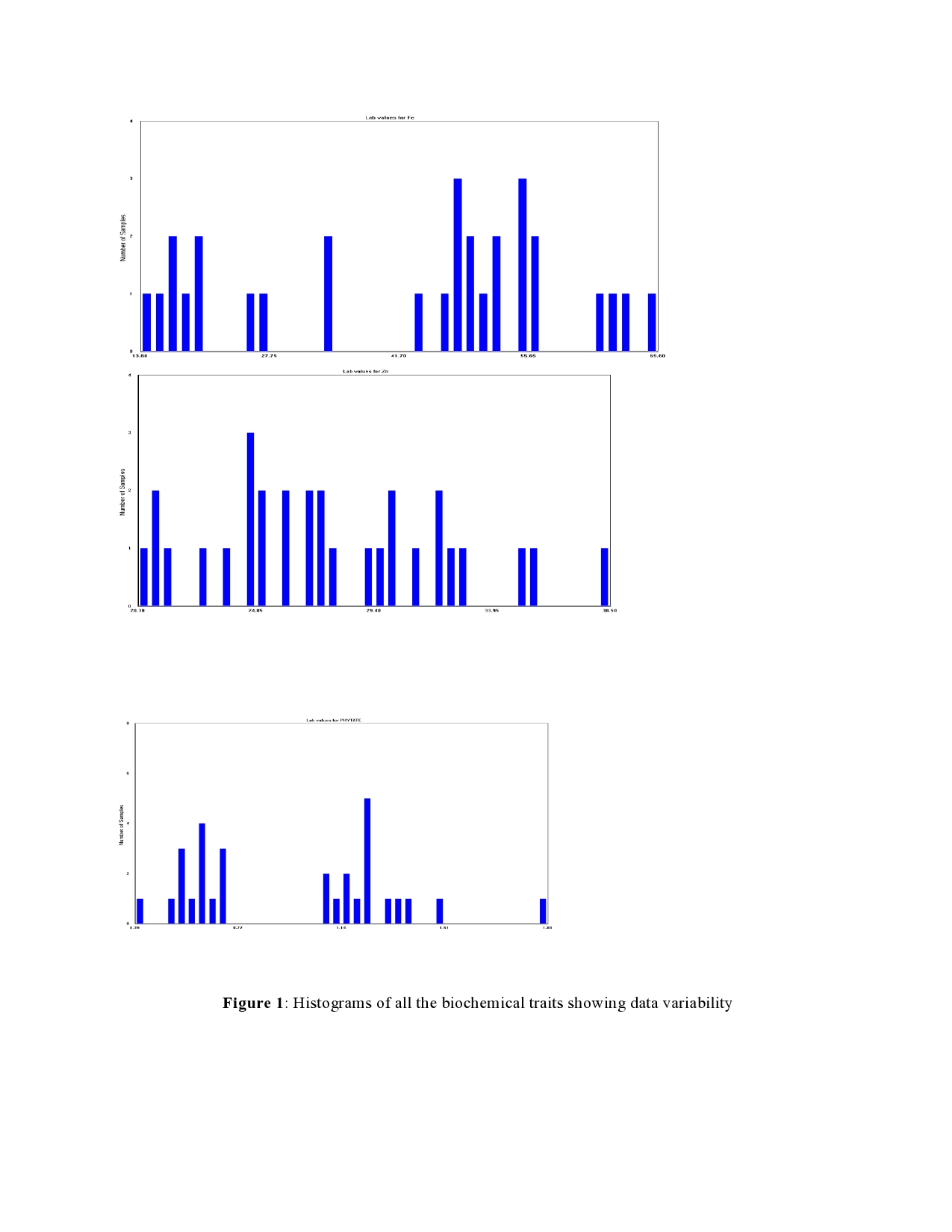

Supplement: Supplementary file 1 [file Data_Sheet_1.ZIP › page0006.jpg]
