# Supplementary material for: Rice bean-adzuki bean multitrait near infrared reflectance spectroscopy prediction model: a rapid mining tool for trait-specific germplasm
Source: Front Nutr. 2023 Dec 15;10:1224955. doi: 10.3389/fnut.2023.1224955 (PMC10757333; doi:10.3389/fnut.2023.1224955)
Supplement: Supplementary file 2 [file Data_Sheet_2.docx]

**Supplementary Table 1: Statistics of calibration sets for different parameters in combined model for adzuki and rice bean genotypes by mPLS methods**

| Parameters | Math  Treatments | Bias | Mean | SECV | No. of Samples |
| --- | --- | --- | --- | --- | --- |
| **Protein** | 2441 | -0.006 | 21.876 | 0.59 | 29 |
| **Dietary Fiber** | 3661 | 0.094 | 16.95 | 1.73 | 28 |
| **Sugar** | 3661 | 0.133 | 4.881 | 0.82 | 24 |
| **Starch** | 3661 | 0.056 | 41.661 | 1.86 | 28 |
| **Phytate** | 3772 | 0 | 0.931 | 0.152 | 29 |
| **Fat** | 3661 | 0.12 | 2.143 | 0.98 | 28 |
| **CUPRAC** | 3772 | -0.048 | 5.972 | 1.02 | 29 |
| **Phenol** | 3 10 10 2 | 0.004 | 0.353 | 0.1149 | 29 |
| **FRAP** | 3661 | 0.012 | 2.527 | 0.82 | 30 |
| **Anthocyanin** | 3661 | -0.097 | 3.011 | 2.97 | 30 |
| **Cu** | 3772 | 0 | 4.786 | 1.53 | 28 |
| **Fe** | 3661 | -0.835 | 41.367 | 14.09 | 27 |
| **Zn** | 2441 | 0.218 | 27.842 | 4.718 | 26 |
| **Ash** | 2441 | -0.002 | 2.692 | 0.39 | 29 |
| **Red** | 3661 | -0.817 | 161.9 | 21.05 | 30 |
| **Green** | 3661 | -1.278 | 124.6 | 20.5 | 30 |
| **Blue** | 3661 | 0.043 | 106.2 | 21.45 | 30 |

**Supplementary Table 2 : Statistics of validation sets for different parameters in combined model for adzuki and rice bean genotypes by mPLS methods**

| Parameters | Math Treatment | Bias | Means | Slope | SEPC | SEDC Limit |
| --- | --- | --- | --- | --- | --- | --- |
| **Protein** | 2441 | 0.079 | 21.908 | 0.872 | 0.323 | 0.769 |
| **Dietary Fiber** | 3661 | 0.436 | 17.45 | 0.957 | 0.964 | 2.249 |
| **Sugar** | 3661 | 0.664 | 5.06 | 0.764 | 0.294 | 1.07 |
| **Starch** | 3661 | 0.274 | 40.839 | 0.768 | 1.294 | 2.429 |
| **Phytate** | 3772 | 0.009 | 0.882 | 0.881 | 0.061 | 0.198 |
| **Fat** | 3661 | -0.327 | 2.072 | 0.341 | 0.482 | 1.277 |
| **CUPRAC** | 3772 | -0.691 | 6.687 | 0.881 | 0.38 | 1.328 |
| **Phenol** | 3 10 10 2 | 0.025 | 0.309 | 0.984 | 0.149 | 0.044 |
| **FRAP** | 3661 | 0.45 | 1.89 | 0.522 | 0.914 | 1.069 |
| **Anthocyanin** | 3661 | 0.365 | 0.731 | 0.52 | 0.758 | 3.87 |
| **Cu** | 3772 | 0.014 | 3.765 | 0.895 | 0.513 | 1.146 |
| **Fe** | 3661 | 1.779 | 47.764 | 0.783 | 10.427 | 18.321 |
| **Zn** | 2441 | -1.132 | 27.361 | 0.547 | 1.615 | 6.135 |
| **Ash** | 2441 | -0.105 | 2.805 | 1.155 | 0.456 | 0.508 |
| **Red** | 4661 | 10.546 | 161.829 | 0.768 | 15.815 | 28.626 |
| **Green** | 4661 | 9.704 | 131.546 | 0.897 | 22.418 | 23.693 |
| **Blue** | 4661 | 6.695 | 120.555 | 0.749 | 15.37 | 27.751 |

**Supplementary Table 3 - Statistics of paired t-Test at 95% confidence level for validation sets under different mathematical treatmentsfor various parameters in combined model for adzuki and rice bean genotypes by mPLS method**

| **Paired Samples Test** | | | | | | | | | | |
| --- | --- | --- | --- | --- | --- | --- | --- | --- | --- | --- |
|  | **Paired Differences** | | | | | | | **t** | **df** | **Sig. (2-tailed)** |
|  | **Mean** | | | **Std. Deviation** | **Std. Error Mean** | **95% Confidence Interval of the Difference** | |  |  |  |
|  |  |  |  |  |  | **Lower** | **Upper** |  |  |  |
| **Pair 1** | | **ProteinLab - ProteinPred** | .079125 | .322733 | .114103 | -.190686 | .348936 | .693 | 9 | .510 |
| **Pair 2** | | **DietaryFiberLab - DietaryFiberPred** | .436000 | .963679 | .364237 | -.455255 | 1.327255 | 1.197 | 9 | .276 |
| **Pair 3** | | **SugarLab - SugarPred** | .664250 | .294065 | .103968 | .418405 | .910095 | 6.389 | 9 | .085 |
| **Pair 4** | | **StarchLab - StarchPred** | .273500 | 1.294019 | .457505 | -.808327 | 1.355327 | .598 | 9 | .569 |
| **Pair 5** | | **PhytateLab - PhytatePred** | .009125 | .060920 | .021539 | -.041806 | .060056 | .424 | 9 | .685 |
| **Pair 6** | | **FatLab - FatPred** | -.326625 | .482453 | .170573 | -.729966 | .076716 | -1.915 | 9 | .097 |
| **Pair 7** | | **AshLab - AshPred** | -.105250 | .456470 | .161386 | -.486868 | .276368 | -.652 | 9 | .535 |
| **Pair 8** | | **FeLab - FePred** | 1.778429 | 10.427172 | 3.941100 | -7.865097 | 11.421954 | .451 | 9 | .668 |
| **Pair 9** | | **CuLab - CuPred** | .013625 | .513494 | .181547 | -.415666 | .442916 | .075 | 9 | .942 |
| **Pair 10** | | **ZnLab - ZnPred** | -1.132143 | 1.614848 | .610355 | -2.625628 | .361342 | -1.855 | 9 | .113 |
| **Pair 11** | | **FRAPLab – FRAPPred** | .4503333 | .9137779 | .3045926 | -.2520585 | 1.1527252 | 1.478 | 9 | .178 |
| **Pair 12** | | **CUPRACLab - CUPRACPred** | -.011125 | .292255 | .103328 | -.255457 | .233207 | -.108 | 9 | .917 |
| **Pair 13** | | **AnthocyaninLab - AnthocyaninPred** | .364857 | .757480 | .286301 | -.335695 | 1.065409 | 1.274 | 9 | .250 |
| **Pair 14** | | **PhenolLab - PhenolPred** | .015250 | .023396 | .008272 | -.004309 | .034809 | 1.844 | 9 | .108 |
| **Pair 15** | | **RedLab - RedPred** | 10.546500 | 15.815253 | 5.591536 | -2.675382 | 23.768382 | 1.886 | 9 | .101 |
| **Pair 16** | | **GreenLab - GreenPred** | 9.704125 | 22.417807 | 7.925892 | -9.037631 | 28.445881 | 1.224 | 9 | .260 |
| **Pair 17** | | **BlueLab - BluePred** | 6.694625 | 15.370230 | 5.434197 | -6.155209 | 19.544459 | 1.232 | 9 | .258 |
